# Nutritional status during hospitalization is associated with the long‐term prognosis of patients with heart failure

**DOI:** 10.1002/ehf2.13629

**Published:** 2021-10-01

**Authors:** Takuma Takada, Kentaro Jujo, Keiko Inagaki, Takuro Abe, Makoto Kishihara, Shota Shirotani, Nana Endo, Shonosuke Watanabe, Kazuhito Suzuki, Yuichiro Minami, Nobuhisa Hagiwara

**Affiliations:** ^1^ Department of Cardiology Tokyo Women's Medical University 8‐1 Kawadacho, Shinjuku‐ku Tokyo 162‐0054 Japan; ^2^ Department of Cardiology Kosei Hospital Tokyo Japan

**Keywords:** Nutritional status, COntrolling NUTritional status score, Heart failure, Cardiovascular event

## Abstract

**Aims:**

The CONtrolling NUTritional status (CONUT) score represents the nutritional status of patients with heart failure (HF). Although high CONUT scores on admission are associated with increased risks of cardiovascular (CV) events in patients with HF, the impact of CONUT changes during hospitalization on their long‐term prognosis is unclear. This study aimed to investigate the impact of CONUT score changes on the clinical outcomes of patients with HF after discharge.

**Methods and results:**

This observational study included 1705 patients hospitalized with HF who were discharged alive. The patients were categorized depending on their CONUT scores at admission and discharge into persistently high, high at admission and normal at discharge, normal at admission and high at discharge, and persistently normal CONUT groups. The primary endpoint was a composite of CV death and readmission for HF after discharge. The primary endpoint occurred in 652 patients (38%) during the median 525 day follow‐up period. Patients with persistently high CONUT scores had the highest composite endpoint rate (log‐rank trend test: *P* < 0.001). After adjusting for covariates, the hazard ratio for the composite outcome was significantly lower for the patients with high CONUT scores at admission and normal CONUT scores at discharge than that for those with persistently high CONUT scores (hazard ratio: 0.69; 95% confidence interval: 0.49–0.98).

**Conclusions:**

Nutritional status changes in patients with HF that occurred during hospitalization were associated with CV events after discharge. Improving the nutritional status of patients may improve their clinical outcomes.

## Introduction

Despite recent medical advances, the clinical outcomes of patients with heart failure (HF) are particularly poor.^1^, ^2^, ^3^ Hospital readmission rates for HF remain high, and they pose a considerable financial burden on healthcare systems.^4^ There is a strong relationship between HF and malnutrition.^5^, ^6^, ^7^, ^8^, ^9^ Regardless of the left ventricular ejection fraction or body weight, the prevalence of malnutrition among patients with HF was >50%.^5^, ^6^ Malnutrition among patients with HF is also related to increased readmission rates for morbidity and mortality associated with cardiovascular (CV) disease.^5^, ^6^, ^7^, ^8^, ^9^ The most severe forms of malnutrition are cardiac cachexia and a catabolic state, which are associated with poor clinical outcomes, because inflammation and neurohormonal activation are augmented.^8^, ^10^, ^11^, ^12^ Therefore, risk stratification requires appropriate evaluations of the nutritional status of patients with HF and especially of aged patients with HF.

The CONtrolling NUTritional status (CONUT) score can identify undernourished patients in hospitalized populations.^13^ A previous study's findings showed that a high CONUT score at baseline was associated with long‐term all‐cause mortality in patients with HF at Stages C and D.^7^, ^9^ Although most adverse events occur after patients are discharged from hospitals, few data describe nutritional assessments of patients with HF at discharge. Furthermore, few studies have focused on the relationships between changes in the nutritional status and the clinical outcomes of patients with HF. Therefore, this study aimed to investigate the impact of CONUT score changes during hospitalization on the clinical outcomes of patients with HF after discharge.

## Methods

### Study population and endpoints

Initially, this study included consecutive patients who were hospitalized for HF at Tokyo Women's Medical University Hospital from July 2013 to September 2019. Patients were diagnosed with HF using the Framingham HF diagnostic criteria.^14^ We excluded patients who died in hospitals, who were lost to follow‐up after discharge, and whose data describing their CONUT scores, at either admission or discharge, were missing. The study population was divided into patients with normal and high CONUT scores at admission; the cut‐off score was 2 points, which was based on a previous report.^13^ The groups were further divided to create four subgroups according to the CONUT scores at discharge using a cut‐off score of 2 points, as follows: (i) persistently high, (ii) high at admission and normal at discharge (high–normal), (iii) normal at admission and high at discharge (normal–high), and (iv) persistently normal. We compared the subgroups' clinical profiles and long‐term prognoses. The study's primary endpoint was a composite of CV death and readmission for HF. CV death included death caused by an acute myocardial infarction, sudden cardiac death, HF, stroke, CV procedures, CV haemorrhage, and other CV events.^15^ The study's protocol was approved by the hospital's ethics committee, and patient enrolment was carried out according to the principles of the Declaration of Helsinki. Written informed consent was obtained from all patients regarding the use of the data from their medical records before study enrolment.

### Data collection and follow‐up

The patients' clinical data at admission and discharge were recorded, including their vital signs, New York Heart Association functional classifications, oral medications, laboratory data [i.e. the complete blood count, haemoglobin, albumin, total bilirubin, blood urea nitrogen (BUN), creatinine, electrolyte, C‐reactive protein, and brain natriuretic peptide (BNP) levels, estimated glomerular filtration rates (eGFRs), and lipid profiles, including the cholesterol and triglyceride levels], and echocardiographic parameters (i.e. the left atrial diameters, left ventricular end‐diastolic diameters, left ventricular ejection fractions, ratios of the early mitral inflow velocity to the early diastolic velocity of the lateral mitral annulus, and right ventricular systolic pressures, which were evaluated during the hospitalization). eGFR was calculated using a previously published formula as follows: eGFR (mL/min/1.73 m^2^) = 194 × serum creatinine^(−1.094)^ × age^(−0.287)^ (× 0.739, if female).^16^ Anaemia was defined as haemoglobin levels of <12.0 g/dL in women and <13.0 g/dL in men.^17^ The groups were compared regarding the aforementioned parameters. After hospital discharge, outpatient appointments were scheduled for at least every 2 months.

### Nutritional assessments

The CONUT scores were calculated from the serum albumin levels, total peripheral lymphocyte counts, and total cholesterol levels, as described previously.^13^ CONUT scores of 0–1 point indicated a normal nutritional status, 2–4 points indicated mild malnutrition, and ≥5 points indicated moderate‐to‐severe malnutrition (Supporting Information, *Table* *S1*). In this study, CONUT scores of 0–1 point were defined as normal CONUT scores, and CONUT scores of ≥2 points were defined as high CONUT scores. Using the Full Nutritional Assessment as the gold standard approach, the CONUT score had a sensitivity of 92.3 and a specificity of 85.0.^13^ Additionally, we determined the geriatric nutritional risk index (GNRI) that was calculated as follows^18^: 1.489 × serum albumin (g/L) + 41.7 × (body weight in kg∕ideal body weight), and the prognostic nutritional index (PNI) that was calculated as follows^19^: 10 × serum albumin (g/dL) + 0.005 × total lymphocyte count (/mm^3^). The body mass index was calculated as the weight (kg)∕[height (m)]^2^. These parameters were assessed at the time of hospital admission and discharge.

### Statistical analyses

The data are expressed as means and standard deviations or as percentiles in the tables. Fisher's exact test was used to evaluate the categorical variables. The Mann–Whitney *U* test was used to compare the continuous variables between two groups. The Kruskal–Wallis test was used to compare the continuous variables among the four groups. The Kaplan–Meier method and log‐rank tests were used to compare the event‐free ratios among the groups during follow‐up. Univariate and multivariable Cox regression analyses were performed to evaluate associations between the baseline characteristics and patient prognoses. Variables were considered clinically significant if they reached a level of significance (*P*) of <0.05, and they were included in the multivariable model. BNP levels were log‐transformed. Another multivariable analysis assessed whether changes in the CONUT scores during hospitalization were associated with the composite endpoint, and the patients' baseline characteristics, namely, age, sex, diabetes mellitus, atrial fibrillation, hypertension, chronic obstructive pulmonary disease, haemodialysis, a history of coronary artery bypass grafting, a family history of heart disease, the left ventricular ejection fraction, and the discharge parameters, namely, the body mass index, heart rate, anaemia, eGFR, C‐reactive protein level, and prescription of angiotensin‐converting enzyme inhibitor/angiotensin receptor blocker, beta‐blocker, or aldosterone antagonist at discharge, were used to adjust the model. Multivariate logistic regression analysis was performed to identify independent factors that could normalize CONUT scores at discharge in patients with high CONUT scores at admission. The factors that were significant in the univariate analysis were also used to adjust the multivariable logistic regression model. A two‐sided *P* value of <0.05 was considered statistically significant. The statistical analyses were performed using R software, Version 3.3.0 (R Foundation for Statistical Computing, Vienna, Austria).

## Results

### Study population

During the study period, 2110 consecutive patients were admitted to our hospital with HF, and 180 (9%) patients died in the hospital (Supporting Information, *Figure* *S1*). Of the 1930 patients who were discharged alive, 180 (9%) whose CONUT scores were missing and 45 (2%) who were lost to follow‐up were excluded. Ultimately, the data of 1705 patients were analysed. The distributions of the study population's CONUT scores at admission and discharge are shown in Supporting Information, *Figure* *S2*. Among the enrolled patients, 1359 (80%) had high CONUT scores, that is, ≥2 points, at admission and 1347 (79%) had high CONUT scores at discharge. Of the patients, 1213 (71%) were in the persistently high, 146 (9%) were in the high–normal, 134 (8%) were in the normal–high, and 212 (12%) were in the persistently normal CONUT groups (Supporting Information, *Figure* *S1*).

### Clinical profiles at admission

*Table* *1* shows the study population's baseline characteristics. Significant differences were evident among the four groups in relation to a diverse range of parameters, except for sex, co‐morbidities associated with diabetes mellitus and chronic obstructive pulmonary disease, the ratio between implantable cardioverter defibrillators and cardiac resynchronization therapy, and the total bilirubin level. The normal–high CONUT group had the highest BNP level at admission. The PNI and GNRI correlated negatively with the CONUT score, and the average values of both indices were within the normal ranges in all groups, even in the persistently high CONUT group (*Table* *1* and Supporting Information, *Table* *S2*). Renin–angiotensin–aldosterone system inhibitors and beta‐blockers were prescribed to >50%, and statins were prescribed to 35% of the entire study population before hospital admission. The subgroups did not differ regarding the prescription rates for renin–angiotensin–aldosterone system inhibitors, beta‐blockers, aldosterone antagonists, inotropes, amiodarone, dipeptidyl peptidase‐4 inhibitors, and sodium–glucose cotransporter‐2 inhibitors. The patients' clinical profiles at discharge are summarized in Supporting Information, *Table* *S3*.

**Table 1:** Baseline characteristics in patients with high or normal CONUT scores at admission or discharge

Variables at admission	Admission–discharge CONUT					*P* value
	All patients	High–high	High–normal	Normal–high	Normal–normal	
	*n* = 1705	*n* = 1213	*n* = 146	*n* = 134	*n* = 212	
Age (years)	71 ± 15	73 ± 14	66 ± 17	70 ± 14	62 ± 15	<0.001
Male	1099 (64%)	785 (65%)	88 (60%)	86 (64%)	140 (66%)	0.39
BMI (kg/m^2^)	23.7 ± 4.7	23 ± 4.5	24 ± 4.9	25 ± 5.1	25 ± 5.2	<0.001
Hypertension	1143 (67%)	822 (68%)	92 (63%)	101 (75%)	128 (60%)	0.020
Diabetes	683 (40%)	503 (41%)	56 (38%)	51 (38%)	73 (34%)	0.24
Insulin‐requiring	196 (11%)	153 (13%)	16 (11%)	16 (12%)	11 (5%)	0.01
Dyslipidaemia	858 (50%)	580 (48%)	75 (51%)	86 (64%)	117 (55%)	0.001
Smoking history	843 (49%)	596 (49%)	68 (47%)	71 (53%)	108 (51%)	0.71
COPD	86 (5%)	71 (6%)	3 (1%)	6 (4%)	6 (3%)	0.086
Family history of IHD	433 (25%)	283 (23%)	42 (29%)	39 (29%)	69 (33%)	0.016
Atrial fibrillation	852 (50%)	655 (54%)	62 (42%)	59 (44%)	76 (36%)	<0.001
Prior PCI	334 (20%)	268 (22%)	21 (14%)	27 (20%)	18 (8%)	<0.001
Prior CABG	138 (8%)	116 (10%)	7 (5%)	11 (8%)	4 (2%)	<0.001
NYHA IV	1258 (74%)	944 (78%)	113 (77%)	97 (72%)	104 (49%)	<0.001
Prior stroke	284 (17%)	224 (18%)	15 (10%)	20 (15%)	25 (12%)	0.011
Haemodialysis	127 (7%)	110 (9%)	4 (3%)	11 (8%)	2 (1%)	<0.001
PM	232 (14%)	187 (15%)	18 (12%)	10 (7%)	17 (8%)	0.003
ICD	235 (14%)	170 (14%)	12 (8%)	21 (16%)	32 (15%)	0.18
CRT	183 (11%)	137 (11%)	11 (8%)	14 (10%)	21 (10%)	0.58
Systolic BP (mmHg)	126 ± 28	125 ± 27	126 ± 26	134 ± 34	126 ± 27	0.052
Diastolic BP (mmHg)	70 ± 18	69 ± 17	74 ± 21	75 ± 22	73 ± 18	<0.001
Heart rate (b.p.m.)	83 ± 22	82 ± 21	87 ± 23	88 ± 28	84 ± 23	0.049
Cardiothoracic ratio (%)	61 ± 7.9	61 ± 8.1	61 ± 7.2	59 ± 6.3	58 ± 7.3	<0.001
Echocardiography						
LVEF (%)	40 ± 13	41 ± 13	38 ± 13	39 ± 13	38 ± 12	0.007
LVDd (mm)	56 ± 11	56 ± 11	58 ± 11	56 ± 10	58 ± 11	<0.001
LAD (mm)	48 ± 11	48 ± 12	48 ± 9.9	46 ± 7.5	45 ± 9.1	<0.001
RVSP (mmHg)	42 ± 14	43 ± 14	39 ± 12	40 ± 13	39 ± 14	<0.001
E/e′	19 ± 9.8	19 ± 10	18 ± 8.7	19 ± 8.5	16 ± 7.3	<0.001
Lab data						
WBC (/μL)	6757 ± 3151	6444 ± 3294	7401 ± 2575	7948 ± 2901	7358 ± 2422	<0.001
Lymphocyte (/μL)	1312 ± 839	1055 ± 599	1360 ± 552	2320 ± 1334	2117 ± 779	<0.001
Haemoglobin (g/dL)	12 ± 2.3	11 ± 2.2	13 ± 2.0	13 ± 2.3	14 ± 2.1	<0.001
Albumin (g/dL)	3.7 ± 0.6	3.6 ± 0.6	3.7 ± 0.5	3.9 ± 0.4	4.2 ± 0.4	<0.001
Total bilirubin (mg/dL)	1.0 ± 0.7	1.1 ± 0.8	1.1 ± 0.7	0.9 ± 0.5	0.9 ± 0.5	0.06
BUN (mg/dL)	30 ± 18	33 ± 20	23 ± 13	30 ± 15	21 ± 13	<0.001
Creatinine (mg/dL)	1.8 ± 1.8	2.0 ± 2.0	1.2 ± 0.6	2.0 ± 2.0	1.1 ± 0.7	<0.001
eGFR (mL/min/1.73 m^2^)	45 ± 31	42 ± 27	53 ± 49	40 ± 20	59 ± 33	<0.001
Sodium (mEq/L)	139 ± 4	139 ± 4.6	140 ± 3.3	140 ± 3.9	140 ± 3.6	<0.001
Potassium (mEq/L)	4.4 ± 0.6	4.4 ± 0.7	4.2 ± 0.4	4.4 ± 0.6	4.2 ± 0.5	<0.001
T‐Chol (mg/dL)	161 ± 40	150 ± 36	172 ± 34	186 ± 34	200 ± 38	<0.001
LDL‐Chol (mg/dL)	90 ± 33	82 ± 29	100 ± 29	107 ± 29	121 ± 33	<0.001
HDL‐Chol (mg/dL)	53 ± 17	52 ± 16	52 ± 19	55 ± 16	55 ± 17	0.02
Triglyceride (mg/dL)	98 ± 63	88 ± 50	108 ± 59	124 ± 102	136 ± 78	<0.001
CRP (mg/dL)	1.43 ± 3.78	1.6 ± 4.3	1.3 ± 2.2	0.9 ± 2.0	0.6 ± 1.8	<0.001
BNP (pg/mL)	908 ± 1053	976 ± 1120	842 ± 678	994 ± 1223	507 ± 535	<0.001
CONUT score at admission	3.5 ± 2.3	4.4 ± 2.0	2.9 ± 1.3	0.7 ± 0.5	0.5 ± 0.5	<0.001
PNI score at admission	44 ± 7.5	41 ± 6.2	44 ± 5.1	51 ± 7.8	53 ± 5.7	<0.001
GNRI score at admission	100 ± 12	98 ± 11	101 ± 11	106 ± 12	110 ± 11	<0.001
Medication at admission						
ACEi/ARB	1046 (61%)	757 (62%)	87 (60%)	85 (63%)	117 (55%)	0.22
Beta‐blocker	956 (56%)	699 (58%)	70 (48%)	79 (59%)	108 (51%)	0.050
Aldosterone antagonist	616 (36%)	447 (37%)	51 (35%)	36 (27%)	82 (39%)	0.11
Thiazide	218 (13%)	175 (14%)	9 (6%)	12 (9%)	22 (10%)	0.008
Furosemide	1043 (61%)	784 (65%)	73 (50%)	68 (51%)	118 (56%)	<0.001
Furosemide dose (mg/day)	37 ± 24	38 ± 25	33 ± 20	35 ± 27	33 ± 19	0.16
Calcium channel blocker	458 (27%)	322 (27%)	38 (26%)	47 (35%)	51 (24%)	0.15
Inotrope	257 (15%)	193 (16%)	17 (12%)	18 (13%)	29 (14%)	0.49
Statin	604 (35%)	458 (38%)	37 (25%)	56 (42%)	53 (25%)	<0.001
Amiodarone	274 (16%)	205 (17%)	16 (11%)	20 (15%)	33 (16%)	0.31
OAC	848 (50%)	652 (54%)	57 (39%)	54 (40%)	85 (40%)	<0.001
SGLT2i	19 (1%)	16 (1%)	0 (0%)	3 (2%)	0 (0%)	0.096

ACEi, angiotensin‐converting enzyme inhibitor; ARB, angiotensin receptor blocker; b.p.m., beats per minute; BMI, body mass index; BNP, brain natriuretic peptide; BP, blood pressure; BUN, blood urea nitrogen; CABG, coronary artery bypass grafting; CONUT, CONtrolling NUTritional status; COPD, chronic obstructive pulmonary disease; CRP, C‐reactive protein; CRT, cardiac resynchronization therapy; E/e′, peak velocity of the early wave (E) to early diastole (e′) ratio; eGFR, estimated glomerular filtration rate; GNRI, geriatric nutritional risk index; HDL, high‐density lipoprotein; ICD, implantable cardioverter defibrillator; IHD, ischemic heart disease; LAD, left atrial dimension; LDL, low‐density lipoprotein; LVDd, left ventricular diastolic diameter; LVEF, left ventricular ejection fraction; NYHA, New York Heart Association functional classification; OAC, oral anticoagulant; PCI, percutaneous coronary intervention; PM, pacemaker; PNI, prognostic nutritional index; RVSP, right ventricular systolic pressure; SGLT2i, sodium–glucose cotransporter‐2 inhibitor; T‐Chol, total cholesterol; WBC, white blood cell.

### Prognosis

**Figure 1:**
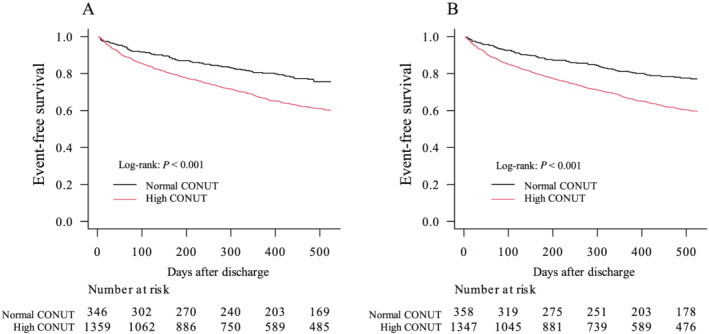
Composite outcome of cardiovascular death and rehospitalization for heart failure after discharge. Comparison of patients with high and normal CONtrolling NUTritional status (CONUT) scores at (A) admission and (B) discharge regarding the composite outcome.

During the median follow‐up duration of 525 days (inter‐quartile range: 295–898 days), of the patients who were discharged alive, 289 patients (14%) died as a consequence of any cause, 165 patients (10%) died as a consequence of CV events, and 591 patients (35%) were readmitted for HF. Overall, composite endpoint events occurred in 652 patients (38%) at a median of 199 days (inter‐quartile range: 65–421 days) after discharge. Patients with high CONUT scores at admission had a significantly higher rate of the primary endpoint than those with normal CONUT scores at admission (log‐rank test: *P* < 0.001) (*Figure* *1* *A*). Cox regression analysis revealed that a CONUT score of ≥2 points at admission remained an independent predictor of the composite endpoint after adjusting for the covariates that were significant in the univariate analysis (*Table* *2*). Compared with the patients with normal CONUT scores at admission, those with high CONUT scores at admission had significantly higher rates of CV death and readmission for HF (log‐rank test: both *P* < 0.001). When the patients were separated into groups with a normal nutritional status (CONUT score: 0–1 point), mild malnutrition (CONUT score: 2–4 points), or moderate‐to‐severe malnutrition (CONUT score: ≥5 points) at admission, the patients with a normal nutritional status showed the lowest composite endpoint rate. The groups of patients with mild malnutrition or moderate‐to‐severe malnutrition did not differ regarding the composite endpoint rate (Supporting Information, *Figure* *S3*). Compared with the patients with CONUT scores of <2 points at discharge, the patients with CONUT scores of ≥2 points had a significantly higher primary endpoint rate (log‐rank test: *P* < 0.001) (*Figure* *1* *B*). The Cox regression analyses that incorporated the discharge parameters consistently showed that a high CONUT score at discharge was associated with the composite endpoint after adjusting for covariates (Supporting Information, *Table* *S4*).

**Table 2:** Cox regression analysis for the composite of cardiovascular death and heart failure readmission using the baseline parameters

Variables	Univariate			Multivariate		
	HR	95% CI	*P* value	HR	95% CI	*P* value
Age	1.02	1.01–1.03	<0.001	1.01	1.00–1.02	0.06
Male	1.04	0.89–1.23	0.60			
BMI	0.95	0.93–0.96	<0.001	0.95	0.93–0.98	<0.001
NYHA IV	1.58	1.31–1.90	<0.001	1.54	1.20–1.99	<0.001
Atrial fibrillation	1.46	1.25–1.71	<0.001	1.11	0.89–1.38	0.33
Diabetes	1.24	1.07–1.45	<0.001	1.34	1.09–1.65	0.006
COPD	1.33	0.96–1.85	0.085			
Haemodialysis	0.88	0.65–1.19	0.41			
Prior CABG	1.42	1.10–1.83	0.006	1.00	0.71–1.39	0.98
Family history of IHD	1.05	0.88–1.30	0.61			
LVEF	0.99	0.99–1.00	0.008	0.99	0.98–1.00	0.24
Systolic BP per 1 mmHg	0.99	0.99–1.00	<0.001	1.00	0.99–1.00	0.050
Heart rate per 1 b.p.m.	0.99	0.99–1.00	<0.001	0.99	0.99–1.00	0.02
Log‐transformed BNP	1.49	1.26–1.76	<0.001	1.15	0.88–1.51	0.30
BUN	1.02	1.01–1.02	<0.001	1.01	1.00–1.02	0.001
eGFR	0.99	0.99–1.00	<0.001	1.00	0.99–1.01	0.87
Anaemia	1.75	1.48–2.06	<0.001	0.97	0.76–1.23	0.78
Sodium	0.95	0.94–0.97	<0.001	0.99	0.97–1.02	0.61
CRP	0.99	0.97–1.02	0.65			
Furosemide dose	1.01	1.00–1.01	<0.001	1.00	1.00–1.01	0.26
Statin	1.11	0.95–1.30	0.20			
High CONUT at admission	1.97	1.58–2.46	<0.001	1.56	1.13–2.16	0.007
ACEi/ARB	1.23	1.09–1.51	0.002	1.03	0.81–1.31	0.78
Beta‐blocker	1.37	1.17–1.61	<0.001	1.07	0.85–1.36	0.55
Aldosterone antagonist	1.70	1.46–1.98	<0.001	1.49	1.19–1.88	<0.001

ACEi, angiotensin‐converting enzyme inhibitor; ARB, angiotensin receptor blocker; b.p.m., beats per minute; BMI, body mass index; BNP, brain natriuretic peptide; BP, blood pressure; BUN, blood urea nitrogen; CABG, coronary artery bypass grafting; CI, confidence interval; CONUT, CONtrolling NUTritional status; COPD, chronic obstructive pulmonary disease; CRP, C‐reactive protein; eGFR, estimated glomerular filtration rate; HR, hazard ratio; IHD, ischemic heart disease; LVEF, left ventricular ejection fraction; NYHA, New York Heart Association functional classification.

In univariate Cox regression analysis, age, BMI, NYHA Class IV, atrial fibrillation, diabetes mellitus, history of CABG, LVEF, systolic BP, heart rate, BNP, eGFR, anaemia, serum sodium, daily furosemide dose, and high CONUT score ≥2 at admission were associated with the incidence of composite endpoint. The prescription of statin at admission and haemodialysis were not statistically related to the composite endpoint. Even after adjusting for significantly related factors in univariate analysis, CONUT score ≥2 at admission remained the independent predictor for the composite endpoint.

### Subgroup comparisons

**Figure 2:**
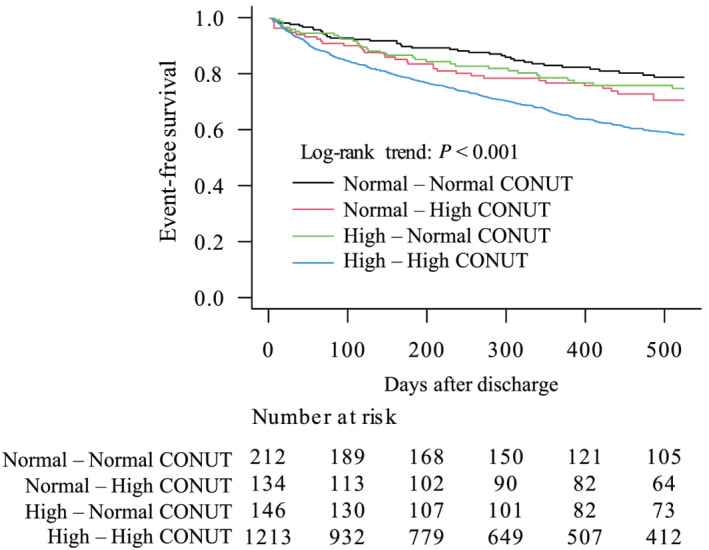
Composite outcome after discharge among the four subgroups categorized according to the CONtrolling NUTritional status (CONUT) scores at admission and discharge. Kaplan–Meier curve of the composite outcome in the four subgroups categorized according to the CONUT scores at admission and discharge using a cut‐off score of 2 points.

During follow‐up, the composite endpoint occurred in 522 patients (43%) in the persistently high, 41 patients (31%) in the normal–high, 39 patients (27%) in the high–normal, and 50 patients (24%) in the persistently normal CONUT groups. The Kaplan–Meier analysis showed that the persistently high CONUT group had the highest rate and the persistently normal CONUT group had the lowest rate of the composite endpoint (log‐rank trend test: *P* < 0.001) (*Figure* *2*). Of the four subgroups, the persistently high CONUT group showed the highest rates of CV death and HF readmission (both *P* < 0.001). After adjusting the model for co‐morbidities and discharge parameters, the persistently high CONUT group and the normal–high CONUT group did not differ in the hazard ratio (HR) for the composite outcome (HR: 0.77; 95% confidence interval: 0.54–1.09; *P* = 0.14), and compared with the persistently high CONUT group, the high–normal CONUT group had a significantly lower HR for the composite outcome (HR: 0.69; 95% confidence interval: 0.49–0.98; *P* = 0.04) (*Table* *3*). When the patients were separated into two groups according to the presence of high and low CONUT scores during the index hospitalization, the primary endpoint rate did not differ between the groups (log‐rank test: *P* = 0.73) (Supporting Information, *Figure* *S4*). Furthermore, when the study population was divided into two groups according to the phenotype of HF, HF with reduced ejection fraction and HF with preserved left ventricular ejection fraction, the patients with normalized CONUT scores had better clinical outcomes than those with persistently high CONUT scores, regardless of the phenotype (Supporting Information, *Figure* *S5*).

**Table 3:** Multivariate analysis by Cox regression of CV death and HF readmission

Variables	Event rate (%)	Age–sex‐adjusted HR	95% CI	*P* value	Multivariate‐adjusted HR	95% CI	*P* value
At admission–discharge							
High–high CONUT	43	1.0			1.0		
Normal–high CONUT	31	0.61	0.45–0.85	0.003	0.77	054–1.09	0.14
High–normal CONUT	27	0.56	0.40–0.77	<0.001	0.69	0.49–0.98	0.04
Normal–normal CONUT	24	0.48	0.35–0.64	<0.001	0.58	0.39–0.86	0.008

ACEi, angiotensin‐converting enzyme inhibitor; ARB, angiotensin receptor blocker; CI, confidence interval; CONUT, CONtrolling NUTritional status; CV, cardiovascular; eGFR, estimated glomerular filtration rate; HF, heart failure; HR, hazard ratio.

Patients' baseline characteristics, namely, age, sex, diabetes mellitus, atrial fibrillation, hypertension, chronic obstructive pulmonary disease, haemodialysis, a history of coronary artery bypass grafting, a family history of heart disease, the left ventricular ejection fraction, and the discharge parameters, namely, the body mass index, heart rate, anaemia, eGFR, and C‐reactive protein level, ACEi/ARB, beta‐blocker, and aldosterone antagonist, were used to adjust the model.

### Predicting normalization of CONtrolling NUTritional status scores at discharge

Logistic regression analysis for the normalization of CONUT scores in patients with high scores at admission revealed that having a younger age, a low BUN level, no anaemia, no statin use, or a low CONUT score at admission was independently associated with the normalization of CONUT scores at discharge (*Table* *4*). In contrast, Supporting Information, *Table* *S5* describes the factors related to changes in the nutritional status from normal CONUT scores at admission to high CONUT scores at discharge. Low eGFR and high CONUT scores at admission were independent predictors for high CONUT scores at discharge in patients with normal CONUT scores at admission (Supporting Information, *Table* *S5*).

**Table 4:** Logistic regression analysis in patients with high CONUT at admission for normal CONUT score at discharge (*n* = 1359)

Variables	Univariate			Multivariate		
	OR	95% CI	*P* value	OR	95% CI	*P* value
Age per 1 year	0.97	0.96–0.98	<0.001	0.98	0.97–1.00	0.04
Male	0.83	0.58–1.18	0.29			
BMI	1.04	1.00–1.08	0.03	1.02	0.98–1.07	0.38
Diabetes mellitus	0.88	0.62–1.25	0.47			
COPD	0.33	0.11–1.09	0.07			
Prior CABG	0.48	0.22–1.04	0.06			
Family history of IHD	1.33	0.91–1.94	0.15			
Atrial fibrillation	0.63	0.65–0.89	0.009	0.70	0.45–1.09	0.11
Haemodialysis	0.28	0.10–0.77	0.01	0.35	0.10–1.22	0.10
NYHA IV	0.98	0.65–1.47	0.91			
LVEF per 1%	0.99	0.97–1.00	0.050	1.01	0.99–1.03	0.38
Systolic BP per 1 mmHg	1.00	0.99–1.01	0.68			
Heart rate per 1 b.p.m.	1.01	1.00–1.02	0.01	1.00	0.99–1.01	0.73
BUN per 1 mg/dL	0.96	0.95–0.97	<0.001	0.97	0.95–0.99	0.01
Log‐transferred BNP	1.16	0.80–1.68	0.45			
eGFR per 1 mL/min/1.73 m^2^	1.01	1.00–1.01	<0.001	1.00	0.99–1.00	0.45
Anaemia	0.23	0.16–0.33	<0.001	0.56	0.36–0.90	0.01
Total bilirubin per 1 mg/dL	1.10	0.89–1.36	0.39			
CRP per 1 mg/dL	0.96	0.90–1.03	0.28			
Sodium per 1 mEq/L	1.07	1.02–1.11	0.004	1.04	0.98–1.10	0.24
Statin	0.56	0.38–0.83	0.004	0.52	0.32–0.85	0.008
Furosemide daily dose per 1 mg	0.99	0.98–1.00	0.13			
CONUT score at admission	0.53	0.45–0.62	<0.001	0.55	0.46–0.67	<0.001

b.p.m., beats per minute; BMI, body mass index; BNP, brain natriuretic peptide; BP, blood pressure; BUN, blood urea nitrogen; CABG, coronary artery bypass grafting; CI, confidence interval; CONUT, CONtrolling NUTritional status; COPD, chronic obstructive pulmonary disease; CRP, C‐reactive protein; eGFR, estimated glomerular filtration rate; IHD, ischemic heart disease; LVEF, left ventricular ejection fraction; NYHA, New York Heart Association functional classification; OR, odds ratio.

## Discussion

### Study findings

This study's findings demonstrated the long‐term clinical prognoses of patients with HF according to changes in their nutritional status during hospitalization. The principal findings were (i) patients with high CONUT scores at admission and discharge had poor long‐term clinical outcomes and (ii) patients with high CONUT scores at admission and normal CONUT scores at discharge had significantly better clinical outcomes than those whose CONUT scores were persistently high.

The current study revealed that the CONUT scores at admission were related to poor clinical outcomes, which is consistent with previous reports.^7^, ^8^, ^9^ Meanwhile, few reports refer to CONUT scores at discharge; Yoshihisa *et al*. showed that high CONUT scores at discharge were associated with all‐cause mortality in patients with HF,^20^ consistent with the current findings. Additionally, our study focused on changes in nutritional status. We have shown that the nutritional status of patients with HF can change during hospitalization and that the patients in subgroups defined according to the CONUT scores at admission and discharge had different clinical profiles and prognoses after discharge, a novel finding.

### Nutritional assessment

We selected the CONUT score to represent the nutritional status of hospitalized patients with HF in the current study, and given that it comprises the results from blood tests only, the CONUT score should be objective and reproducible. In addition, the CONUT score accurately represents a patient's nutritional status, and it predicts short‐ and long‐term prognoses, because the albumin levels and lymphocyte counts may be associated with a patient's prognosis at different time points.^21^

The PNI and GNRI correlated negatively with the CONUT score, but the PNI and GNRI at admission were within their normal ranges when the CONUT score was abnormal (Supporting Information, *Table* *S2*). A lower GNRI at admission is associated with higher in‐hospital and long‐term mortality rates in patients hospitalized with HF.^22^, ^23^ Although the GNRI is useful for prognostic risk stratification, patients' body weights cannot always be determined during the acute phase of HF because the patients are intubated, their vital signs are monitored continuously, and mechanical circulatory support systems are used. Unlike the CONUT score, the PNI does not account for the total cholesterol level, and given that the total cholesterol level is an established long‐term prognostic predictor,^21^ the PNI might be of less prognostic value than the CONUT scoring system in relation to long‐term outcomes. Additionally, the PNI might underestimate a slightly malnourished patient's status, because it does not include criteria that account for mild malnutrition.^5^ The CONUT score may reflect low plasma cholesterol levels resulting from statin therapy,^5^ yet when statin treatment was considered in the current study, the CONUT score remained an independent predictor of the long‐term prognoses of patients hospitalized with HF. Classifying the patients into three strata according to their CONUT scores at admission showed that the risk of the composite endpoint was the lowest for the patients whose nutritional status was normal and that the risk of the composite endpoint was comparable for the patients with mild or moderate‐to‐severe malnutrition (Supporting Information, *Figure* *S3*). Therefore, these findings show that mild malnutrition should not be overlooked, and they suggest that stratifying patients according to risk using a cut‐off score of 2 points may be acceptable for assessing the prognoses of patients with HF.

Patients with high CONUT scores at admission had worse CV prognoses after discharge than those with normal CONUT scores at admission, and within this high‐risk population, the patients in the persistently high CONUT group had worse prognoses than the patients in the high–normal CONUT group, regardless HF phenotype. We expected the patients in the persistently normal and the high–normal CONUT groups to have better clinical outcomes. Surprisingly, the patients in the normal–high and persistently high CONUT groups had similar prognoses; the normal–high CONUT group had the highest BNP level at admission, which may have influenced these results. Meanwhile, increases or decreases in the CONUT scores during hospitalization were not associated with the prognosis. Hence, patients with HF must be stratified according to the presence or absence of malnutrition at admission, and if malnutrition is evident, it is also important to check for improvements during hospitalization.

### Clinical implications

Regarding the characteristics of patients whose nutritional status may improve at discharge, the multivariate logistic regression analysis showed that absence of anaemia, lower BUN levels, no statin use, and lower CONUT scores at admission were independent predictors of a normal CONUT score at discharge. Anaemia and high BUN levels are also adversely associated with mortality among patients with HF,^21^, ^24^, ^25^ consistent with the current findings. Whether these factors cause malnutrition is unclear, but the PNI and CONUT scores correlate with the haemoglobin level at admission.^8^, ^26^ We considered that to improve anaemia, it was necessary to detect the appropriate cause of anaemia, including ferritin levels; the insufficient erythropoiesis with chronic kidney disease; or the deficiency of iron, copper, zinc, pyridoxine, tocopherol, cobalamin, folic acid, or ascorbic acid, which were also generally related to the nutritional status. However, these results could indicate that patients with both HF and these characteristics at admission were experiencing cachexia or their general condition was poor, because anaemia is included in the definition of cachexia.^27^ Regarding statin use, two large, randomized studies' findings have shown that statins are not associated with better clinical outcomes in patients with HF.^28^, ^29^ In addition, some guidelines do not primarily recommend statin therapy for patients with HF.^30^ Therefore, if malnourished patients with HF have begun statin therapy, the timing of the statin prescription should be evaluated carefully.

Although an appropriate strategy for improving a patient's nutritional status remains unclear, this study's findings suggest that if it is poor at admission and it improves to a normal nutritional status when the patient is discharged from hospital, their prognosis will be ameliorated. There are no standard protocols for improving nutritional status because each patient's nutritional status and background vary. However, nutritional assessment, counselling, and education are performed at various points by the nutrition support team (NST) in Japan during the index hospitalization, during which the patients' nutritional status, severity of HF, frailty, age, family support environment or nursing facility, and swallowing and mastication function are assessed. The NST comprehensively decides the appropriate food form or stiffness as well as the optimal food administration route (i.e. oral, intravenous, or tube feeding). Recently, Hersberger *et al*. demonstrated that individual nutritional support for hospitalized patients with chronic HF improved their mortality.^31^ Therefore, we should carefully assess the nutritional status of patients at admission, strive to improve their nutritional status, and re‐evaluate their nutritional status at discharge. Additionally, a multidisciplinary approach that involves nutritionists is crucial to achieve better clinical outcomes.

### Limitations

This was a retrospective study performed in a single centre. We did not evaluate the patients' muscle volumes, exercise capacities, fatty acid metabolisms, iron dynamics, or sarcopenia. Although some patients received nutritional assessment and counselling through the NST during hospitalization, there were no data regarding their nutritional status or compliance after discharge. To date, very few publications have described nutritional interventions that can improve the prognoses of patients with HF.^32^ Indeed, some factors such as age, BUN level, anaemia, and CONUT score at admission were associated with an improvement in malnutrition at discharge; however, we could not propose the effect of these factors on malnutrition or an approach that would improve the nutritional status of a patient and reduce the CONUT score based on the findings from this study. A change in the CONUT score might only represent the nutritional status of the patients who have reserves for prognostic improvement. Although we grouped the patients according to their CONUT scores using a cut‐off score of 2 points to ensure we did not miss minor malnutrition, other cut‐off values were not examined. Further large prospective investigations are needed to determine the best management strategy for patients with HF.

## Conclusions

Changes in the nutritional status of patients with HF during hospitalization were associated with CV death or HF readmission after discharge. To stratify patients with HF appropriately according to risk, their nutritional status must be re‐evaluated after initial treatment, and nutritional interventions should be considered for this refractory population.

## Conflict of interest

None declared.

## Funding

This study was not supported financially by any company, grant, or fund.

## Supporting information


**Table S1.** Assessment of malnutrition by Controlling Nutritional Status (CONUT) score.
**Table S2.** Nutritional status at admission in patients with persistently high CONUT score during hospitalization.
**Table S3.** Patients profile at discharge in high or normal CONUT at admission and discharge.
**Table S4.** Cox regression analysis for the composite of cardiovascular death and heart failure readmission using the parameters at discharge.
**Table S5.** Logistic regression analysis in patients with normal CONUT at admission for high CONUT score at discharge (n = 346).
**Figure S1.** Study population CONUT = CONtrolling NUTritional status; HF = heart failure.
**Figure S2.** Distribution of CONUT score Number of patients at each CONUT score at admission (blue) and discharge (red). CONUT = controlling nutritional status.
**Figure S3.** Combined outcome after discharge among 3 subgroups classified with the nutritional status at admission.
**Note:** Division of the study population into three groups with normal nutritional status (CONUT 0–1 points), mild malnutrition (CONUT score 2–4 points) and moderate to severe malnutrition (CONUT score ≥ 5 points) at admission. Ad = admission; CONUT = controlling nutritional status.
**Figure S4.** Combined outcome after discharge between patients with the raising and lowering of CONUT score during the index hospitalization.
**Note:** All patients were divided into the two groups by the difference between CONUT score at admission and discharge; ΔCONUT = CONUT score at discharge – CONUT score at admission.
CONUT = controlling nutritional status.
**Figure S5.** Composite outcome after discharge among the 4 subgroups categorized according to the CONUT scores at admission and discharge in patients with HFrEF or HFpEF
CONUT = controlling nutritional status; HFpEF = heart failure with preserved left ventricular ejection fraction; HFrEF = heart failure with reduced ejection fraction.
