# Supplementary material for: Nutritional status during hospitalization is associated with the long‐term prognosis of patients with heart failure
Source: ESC Heart Fail. 2021 Oct 1;8(6):5372–82. doi: 10.1002/ehf2.13629 (PMC8712841; doi:10.1002/ehf2.13629)
Supplement: Supplementary file 1 — Table S1. Assessment of malnutrition by Controlling Nutritional Status (CONUT) score. Table S2. Nutritional status at admission in patients with persistently high CONUT score during hospitalization. Table S3. Patients profile at discharge in high or normal CONUT at admission and discharge. Table S4. Cox regression analysis for the composite of cardiovascular death and heart failure readmission using the parameters at discharge. Table S5. Logistic regression analysis in patients with normal CONUT at admission for high CONUT score at discharge (n = 346). Figure S1. Study population CONUT = CONtrolling NUTritional status; HF = heart failure. Figure S2. Distribution of CONUT score Number of patients at each CONUT score at admission (blue) and discharge (red). CONUT = controlling nutritional status. Figure S3. Combined outcome after discharge among 3 subgroups classified with the nutritional status at admission. Note: Division of the study population into three groups with normal nutritional status (CONUT 0–1 points), mild malnutrition (CONUT score 2–4 points) and moderate to severe malnutrition (CONUT score ≥ 5 points) at admission. Ad = admission; CONUT = controlling nutritional status. Figure S4. Combined outcome after discharge between patients with the raising and lowering of CONUT score during the index hospitalization. Note: All patients were divided into the two groups by the difference between CONUT score at admission and discharge; ΔCONUT = CONUT score at discharge – CONUT score at admission. CONUT = controlling nutritional status. Figure S5. Composite outcome after discharge among the 4 subgroups categorized according to the CONUT scores at admission and discharge in patients with HFrEF or HFpEF CONUT = controlling nutritional status; HFpEF = heart failure with preserved left ventricular ejection fraction; HFrEF = heart failure with reduced ejection fraction. [file EHF2-8-5372-s001.docx]

**Supporting Information**

**Supplementary Tables**

**Table S1. Assessment of malnutrition by Controlling Nutritional Status (CONUT) score**

| **Parameters** | **Normal nutrition** | **Mild malnutrition** | **Moderate malnutrition** | **Severe malnutrition** |
| --- | --- | --- | --- | --- |
| **Serum Albumin (g/dL)** | ≧ 3.5 | 3.0-3.49 | 2.5-2.9 | ＜2.5 |
| **Serum Albumin Score** | 0 | 2 | 4 | 6 |
| **Total Lymphocytes/μL** | ≧1,600 | 120-1599 | 800-1199 | ＜800 |
| **Total Lymphocytes Score** | 0 | 1 | 2 | 3 |
| **Total Cholesterol (mg/dL)** | ≧180 | 140-180 | 100-139 | ＜100 |
| **Total Cholesterol Score** | 0 | 1 | 2 | 3 |
| **Screening Total Score** | **0-1** | **2-4** | **5-8** | **9-12** |

The CONUT score was calculated from the serum albumin levels, total peripheral lymphocyte counts, and total cholesterol levels. Early detection of hospital malnutrition using CONUT score was sensitivity of 92.3% and specificity of 85.0% (13).

**Table S2. Nutritional status at admission in patients with persistently high CONUT score during hospitalization**

|  | **Persistently high CONUT patients (High-High CONOUT score)** | | | |
| --- | --- | --- | --- | --- |
|  | **Normal nutrition** | **Mild malnutrition** | **Moderate malnutrition** | **Severe malnutrition** |
| **CONUT at admission** | 0% | 61% | 34% | 5% |
| **GNRI at admission** | 50% | 20% | 22% | 8% |
| **PNI at admission** | 71% | NA | 14% | 15% |

CONUT score of 0-1, 2-4, 5-8, and 9-12 indicates normal nutrition, mild, moderate, and severe malnutrition, respectively.

GNRI score of >98, 92-97, 82-91, and <82 indicates normal nutrition, mild, moderate, and severe malnutrition, respectively

PNI score of >38, 35 to 38 and <35 indicates normal nutrition, moderate and severe malnutrition, respectively. There is no “mild” category for PNI.

CONUT = controlling nutritional status; GNRI = geriatric nutritional risk index; PNI = prognostic nutritional index

**Table S3. Patients profile at discharge in high or normal CONUT at admission and discharge**

| **Variables at discharge** |  |  | **Admission-Discharge CONUT** | | | | **p Value** |
| --- | --- | --- | --- | --- | --- | --- | --- |
|  | **All patients** |  | **High-High** | **High-Normal** | **Normal-High** | **Normal-Normal** |  |
|  | **n = 1,705** |  | **n = 1,213** | **n = 146** | **n = 134** | **n = 212** |  |
| BMI, kg/m^2^ | 22 ± 4.4 |  | 21 ± 4.1 | 22 ± 4.2 | 23 ± 4.7 | 24 ± 4.6 | < 0.001 |
| Systolic BP, mmHg | 112 ± 19 |  | 112 ± 19 | 110 ± 17 | 116 ± 19 | 112 ± 18 | 0.06 |
| Diastolic BP, mmHg | 61 ± 10 |  | 60 ± 10 | 63 ± 10 | 63 ± 11 | 63 ± 10 | < 0.001 |
| Heart rate, bpm | 71 ± 12 |  | 71 ± 13 | 72 ± 13 | 69 ± 11 | 69 ± 12 | 0.03 |
| Lab data |  |  |  |  |  |  |  |
| WBC, /uL | 5,681 ± 2,709 |  | 5,521 ± 3,019 | 6,049 ± 1,639 | 5,987 ± 1,786 | 6,154 ± 1,589 | < 0.001 |
| Lymphocyte, /uL | 1349 ± 784 |  | 1,179 ± 810 | 1,830 ± 429 | 1,446 ± 512 | 1,929 ± 492 | < 0.001 |
| Hemoglobin, g/dL | 12 ± 2.2 |  | 12 ± 2.0 | 13 ± 1.9 | 12 ± 2.0 | 14 ± 2.0 | < 0.001 |
| Albumin, g/dL | 3.6 ± 0.5 |  | 3.5 ± 0.5 | 3.9 ± 0.3 | 3.6 ± 0.4 | 4.1 ± 0.4 | < 0.001 |
| Total bilirubin, mg/dL | 0.9 ± 0.6 |  | 0.9 ± 0.6 | 0.9 ± 0.4 | 0.8 ± 0.4 | 0.8 ± 0.4 | 0.32 |
| BUN, mg/dL | 31 ± 17 |  | 33 ± 18 | 26 ± 15 | 28 ± 14 | 23 ± 13 | < 0.001 |
| Creatinine, mg/dL | 1.8 ± 2.0 |  | 2.0 ± 2.2 | 1.2 ± 0.7 | 1.9 ± 2.0 | 1.1 ± 0.7 | < 0.001 |
| eGFR, mL/min/1.73m^2^ | 46 ± 32 |  | 43 ± 26 | 56 ± 58 | 41 ± 21 | 59 ± 37 | < 0.001 |
| Sodium, mEq/L | 139 ± 4 |  | 138 ± 3.8 | 139 ± 3.1 | 139 ± 3.5 | 139 ± 3.0 | < 0.001 |
| T-Chol, mg/dL | 159± 38 |  | 149 ± 35 | 181 ± 28 | 174 ± 35 | 194 ± 32 | < 0.001 |
| CRP, mg/dL | 0.88 ± 1.7 |  | 0.96 ± 1.87 | 0.44 ± 0.68 | 1.23 ± 2.12 | 0.51 ± 0.88 | < 0.001 |
| BNP, pg/mL | 465 ± 648 |  | 531 ± 730 | 268 ± 229 | 489 ± 491 | 222 ± 235 | < 0.001 |
| CONUT score at discharge | 3.4 ± 2.2 |  | 4.3 ± 2.0 | 0.8 ± 0.4 | 3.0 ± 1.1 | 0.6 ± 0.5 | < 0.001 |
| PNI score at discharge | 43 ± 6.9 |  | 41 ± 6.5 | 48 ± 3.9 | 43 ± 4.4 | 50 ± 4.4 | < 0.001 |
| GNRI score at discharge | 100 ± 15 |  | 93 ± 11 | 101 ± 10 | 98 ± 12 | 106 ± 10 | < 0.001 |
| Medication at discharge |  |  |  |  |  |  |  |
| ACEi/ARB | 1,338 (78%) |  | 917 (76%) | 123 (84%) | 112 (84%) | 186 (88%) | < 0.001 |
| Beta blocker | 1,315 (77%) |  | 901 (74%) | 125 (86%) | 115 (86%) | 174 (82%) | < 0.001 |
| Aldosterone antagonist | 974 (57%) |  | 661 (54%) | 103 (71%) | 74 (55%) | 136 (64%) | < 0.001 |
| Thiazide | 222 (13%) |  | 179 (15%) | 11 (8%) | 10 (7%) | 22 (10%) | 0.006 |
| Furosemide | 1,418 (83%) |  | 1,031 (85%) | 123 (84%) | 99 (74%) | 165 (78%) | 0.002 |
| Furosemide dose, mg/day | 37 ± 27 |  | 39 ± 28 | 29 ± 17 | 34 ± 25 | 32 ± 25 | < 0.001 |
| Calcium channel blocker | 410 (24%) |  | 301 (25%) | 29 (20%) | 39 (29%) | 41 (19%) | 0.10 |
| Inotrope | 275 (16%) |  | 199 (16%) | 25 (17%) | 19 (14%) | 328 (15%) | 0.89 |
| Statin | 721 (42%) |  | 513 (42%) | 51 (35%) | 70 (52%) | 87 (41%) | 0.03 |
| Amiodarone | 363 (21%) |  | 255 (21%) | 28 (19%) | 29 (22%) | 51 (24%) | 0.70 |
| OAC | 989 (58%) |  | 739 (61%) | 79 (54%) | 71 (53%) | 100 (47%) | < 0.001 |
| SGLT2i | 31 (2%) |  | 24 (2%) | 1 (1%) | 5 (4%) | 1 (0%) | 0.11 |

ACEi = angiotensin converting enzyme inhibitor; ARB = angiotensin receptor blocker; BMI = body mass index; BNP = brain natriuretic peptide; BP = blood pressure; bpm = beats per minute; BUN = blood urea nitrogen; CONUT = controlling nutritional status; CRP = C-reactive protein; GNRI = geriatric nutritional risk index; PNI = prognostic nutritional index; OAC = oral anticoagulants; SGLT2i = sodium-glucose cotranporter-2 inhibitor; WBC = white blood cell

**Table S4. Cox regression analysis for the composite of cardiovascular death and heart failure readmission using the parameters at discharge**

|  |  |  |  |  |  |  |  |
| --- | --- | --- | --- | --- | --- | --- | --- |
| **Variables** | **Univariate** | | |  | **Multivariate** | | |
|  | **HR** | **95% CI** | **p Value** |  | **HR** | **95% CI** | **p Value** |
| BMI at discharge | 0.95 | 0.93 - 0.96 | < 0.001 |  | 0.98 | 0.95 - 1.00 | 0.10 |
| Systolic BP at discharge, per 1 mmHg | 0.99 | 0.98 - 0.99 | < 0.001 |  | 0.99 | 0.99 - 1.00 | 0.005 |
| Heart rate at discharge, per 1 bpm | 1.00 | 0.99 - 1.01 | 0.71 |  |  |  |  |
| Log-transformed BNP at discharge | 1.96 | 1.62 - 2.38 | < 0.001 |  | 1.67 | 1.31 - 2.10 | < 0.001 |
| BUN at discharge | 1.02 | 1.01 - 1.02 | < 0.001 |  | 1.01 | 1.00 - 1.02 | 0.047 |
| eGFR at discharge | 0.99 | 0.99 - 1.00 | < 0.001 |  | 1.00 | 0.99 - 1.01 | 0.54 |
| Anemia at discharge | 1.63 | 1.88 - 1.93 | < 0.001 |  | 1.31 | 1.03 - 1.66 | 0.03 |
| Sodium at discharge | 0.94 | 0.92 - 0.96 | < 0.001 |  | 0.95 | 0.93 - 0.98 | < 0.001 |
| CRP at discharge | 0.99 | 0.93 - 1.04 | 0.64 |  |  |  |  |
| Furosemide dose at discharge | 1.01 | 1.00 - 1.01 | < 0.001 |  | 1.00 | 0.99 - 1.01 | 0.27 |
| Statin at discharge | 1.02 | 0.88 – 1.19 | 0.79 |  |  |  |  |
| High CONUT at discharge | 2.11 | 1.69 - 2.64 | < 0.001 |  | 1.48 | 1.09 - 2.01 | 0.01 |
| ACEi/ARB | 0.65 | 0.55 - 0.78 | < 0.001 |  | 0.72 | 0.56 - 0.92 | 0.008 |
| Beta blocker | 0.84 | 0.70 - 1.00 | 0.051 |  |  |  |  |
| Aldosterone antagonist | 1.01 | 0.87 - 1.19 | 0.86 |  |  |  |  |
|  |  |  |  |  |  |  |  |

ACEi = angiotensin converting enzyme inhibitor; ARB = angiotensin receptor blocker; BMI = body mass index; BNP = brain natriuretic peptide; BP = blood pressure; bpm = beats per minute; BUN = blood urea nitrogen; Cl = confidence interval; CONUT = controlling nutritional status; CRP = C-reactive protein; eGFR = estimated glomerular filtration rate; HR = hazard ratio

**Table S5. Logistic regression analysis in patients with normal CONUT at admission for high CONUT score at discharge (n = 346)**

|  |  |  |  |  |  |  |  |
| --- | --- | --- | --- | --- | --- | --- | --- |
| **Variables** | **Univariate** | | |  | **Multivariate** | | |
|  | **OR** | **95% CI** | **p Value** |  | **OR** | **95% CI** | **p Value** |
| Age, per 1 year | 1.04 | 1.02 - 1.05 | <0.001 |  | 1.01 | 0.99 - 1.03 | 0.34 |
| Male | 0.92 | 0.59 - 1.45 | 0.72 |  |  |  |  |
| BMI | 0.98 | 0.94 - 1.03 | 0.41 |  |  |  |  |
| Diabetes mellitus | 1.17 | 0.75 - 1.83 | 0.49 |  |  |  |  |
| COPD | 1.61 | 0.51 - 5.10 | 0.42 |  |  |  |  |
| Prior CABG | 4.65 | 1.45 - 14.9 | 0.01 |  | 3.24 | 0.77 - 13.7 | 0.11 |
| Family history | 0.85 | 0.53 - 1.36 | 0.50 |  |  |  |  |
| Atrial fibrillation | 1.41 | 0.91 - 2.19 | 0.13 |  |  |  |  |
| Hemodialysis | 9.39 | 2.05 - 43.1 | 0.004 |  | 3.13 | 0.53 - 18.6 | 0.21 |
| NYHA 4 | 2.72 | 1.71 – 4.33 | <0.001 |  | 1.72 | 0.93 - 3.19 | 0.09 |
| LVEF, per 1% | 1.01 | 0.99 - 1.02 | 0.51 |  |  |  |  |
| Systolic BP, per 1 mmHg | 1.01 | 1.00 - 1.02 | 0.01 |  | 1.00 | 0.99 - 1.01 | 0.31 |
| Heart rate, per 1 bpm | 1.01 | 0.99 - 1.02 | 0.09 |  |  |  |  |
| BUN, per 1 mg/dL | 1.04 | 1.03 - 1.06 | <0.001 |  | 1.01 | 0.98 - 1.03 | 0.64 |
| Log-transferred BNP | 2.81 | 1.75 - 4.53 | <0.001 |  |  |  |  |
| eGFR, per 1 mL/min/1.73m^2^ | 0.96 | 0.95 - 0.97 | <0.001 |  | 0.97 | 0.96 - 0.99 | 0.009 |
| Anemia | 2.68 | 1.67 - 4.29 | <0.001 |  | 0.92 | 0.49 - 1.71 | 0.79 |
| Total bilirubin, per 1 mg/dL | 1.02 | 0.64 - 1.62 | 0.93 |  |  |  |  |
| CRP, per 1 mg/dL | 1.01 | 0.96 - 1.25 | 0.16 |  |  |  |  |
| Sodium, per 1 mEq/L | 0.97 | 0.92 - 1.03 | 0.38 |  |  |  |  |
| Statin | 2.15 | 1.36 - 3.42 | 0.001 |  | 1.29 | 0.73 - 2.27 | 0.38 |
| Furosemide daily dose, per 1 mg | 1.00 | 0.99 - 1.02 | 0.65 |  |  |  |  |
| CONUT score at admission | 2.48 | 1.56 - 3.94 | <0.001 |  | 2.49 | 1.44 - 4.30 | 0.001 |
|  |  |  |  |  |  |  |  |

**BMI = body mass index; BNP = brain natriuretic peptide; BP = blood pressure; bpm = beats per minute; BUN = blood urea nitrogen; CABG = coronary artery bypass grafting; CI = confidence interval; CONUT = controlling nutritional status; COPD = chronic obstructive pulmonary disease; CRP = C-reactive protein; eGFR = estimated glomerular filtration rate; HT = hypertension; LVEF = left ventricular ejection fraction; NYHA = New York Heart Association functional classification; OR = odds ratio**

**Supplementary Figure Legends**

**Figure S1. Study population**

CONUT = CONtrolling NUTritional status; HF = heart failure.

**Figure S2. Distribution of CONUT score**

Number of patients at each CONUT score at admission (blue) and discharge (red).

CONUT = controlling nutritional status

**Figure S3.** **Combined outcome after discharge among 3 subgroups classified with the nutritional status at admission**

Note: Division of the study population into three groups with normal nutritional status (CONUT 0–1 points), mild malnutrition (CONUT score 2–4 points) and moderate to severe malnutrition (CONUT score ≥ 5 points) at admission.

Ad = admission; CONUT = controlling nutritional status

**Figure S4.** **Combined outcome after discharge between patients with the raising and lowering of CONUT score during the index hospitalization**

Note: All patients were divided into the two groups by the difference between CONUT score at admission and discharge; ΔCONUT = CONUT score at discharge – CONUT score at admission.

CONUT = controlling nutritional status

**Figure S5. Composite outcome after discharge among the 4 subgroups categorized according to the CONUT scores at admission and discharge in patients with HFrEF or HFpEF**

CONUT = controlling nutritional status; HFpEF = heart failure with preserved left ventricular ejection fraction; HFrEF = heart failure with reduced ejection fraction

**Supplementary Figures**

**Figure S1. Study Population**

**Figure S2. Distribution of CONUT score**

**
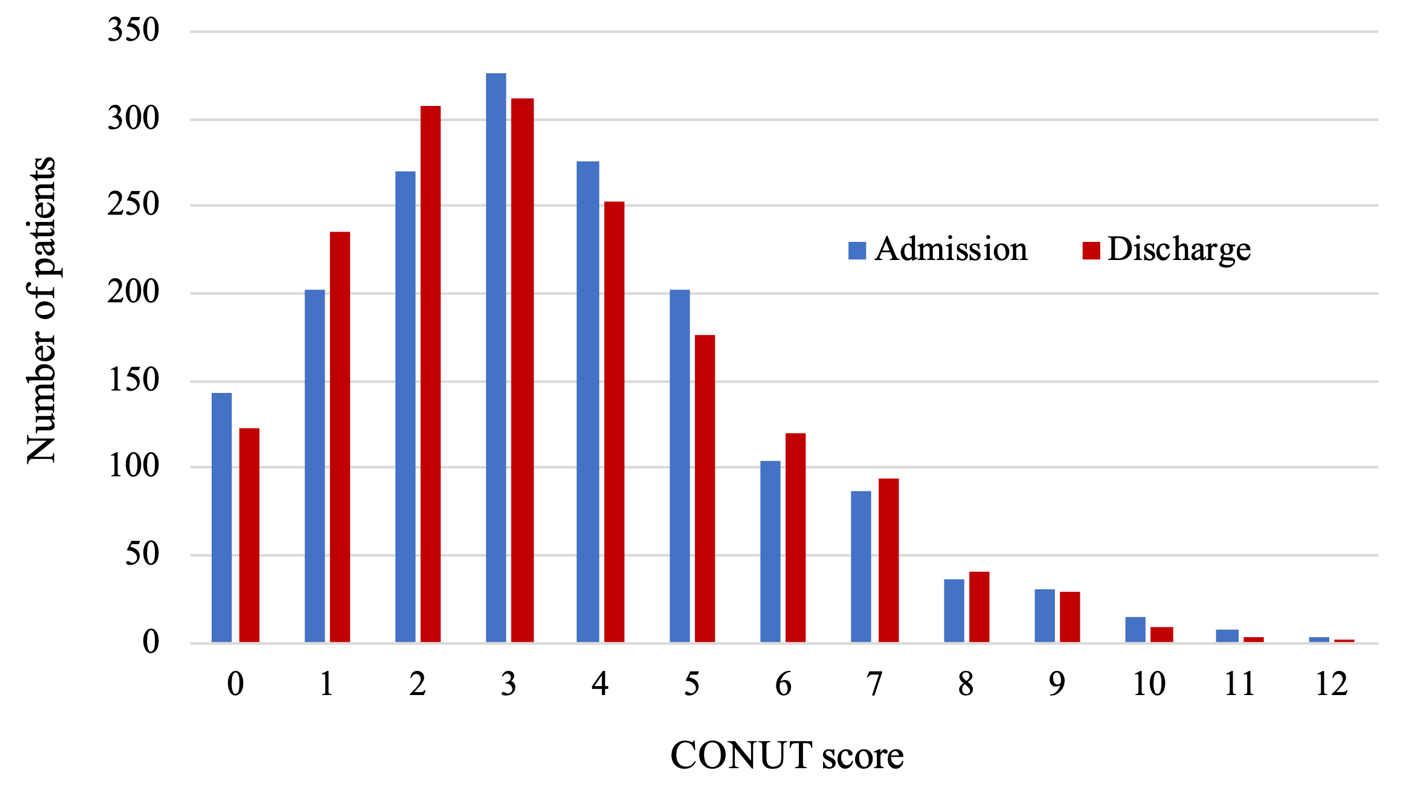
**

**Figure S3. Combined outcome after discharge among 3 subgroups classified with the nutritional status at admission**

**
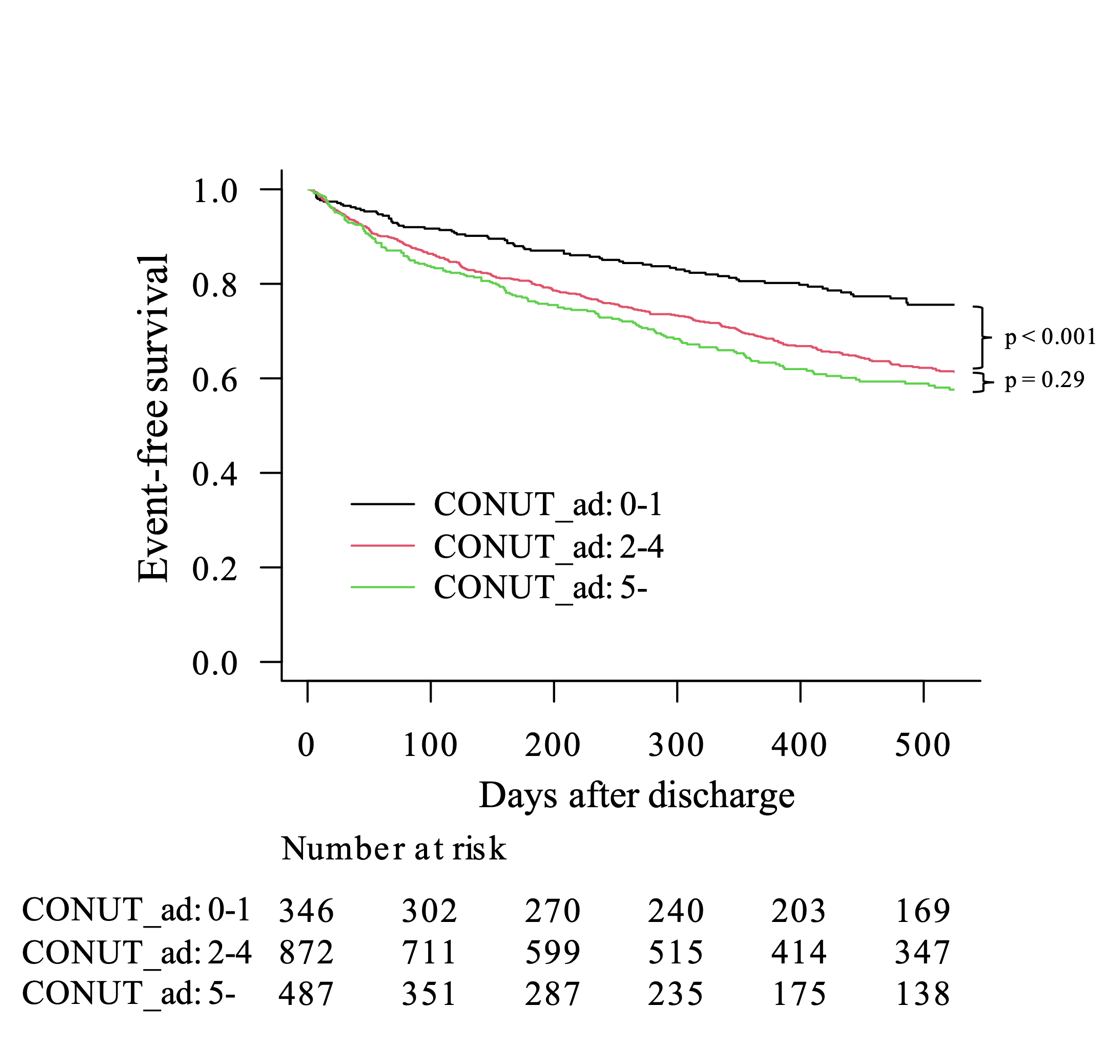
**

**Figure S4. Combined outcome after discharge between patients with the raising and lowering of CONUT score during the index hospitalization**

**
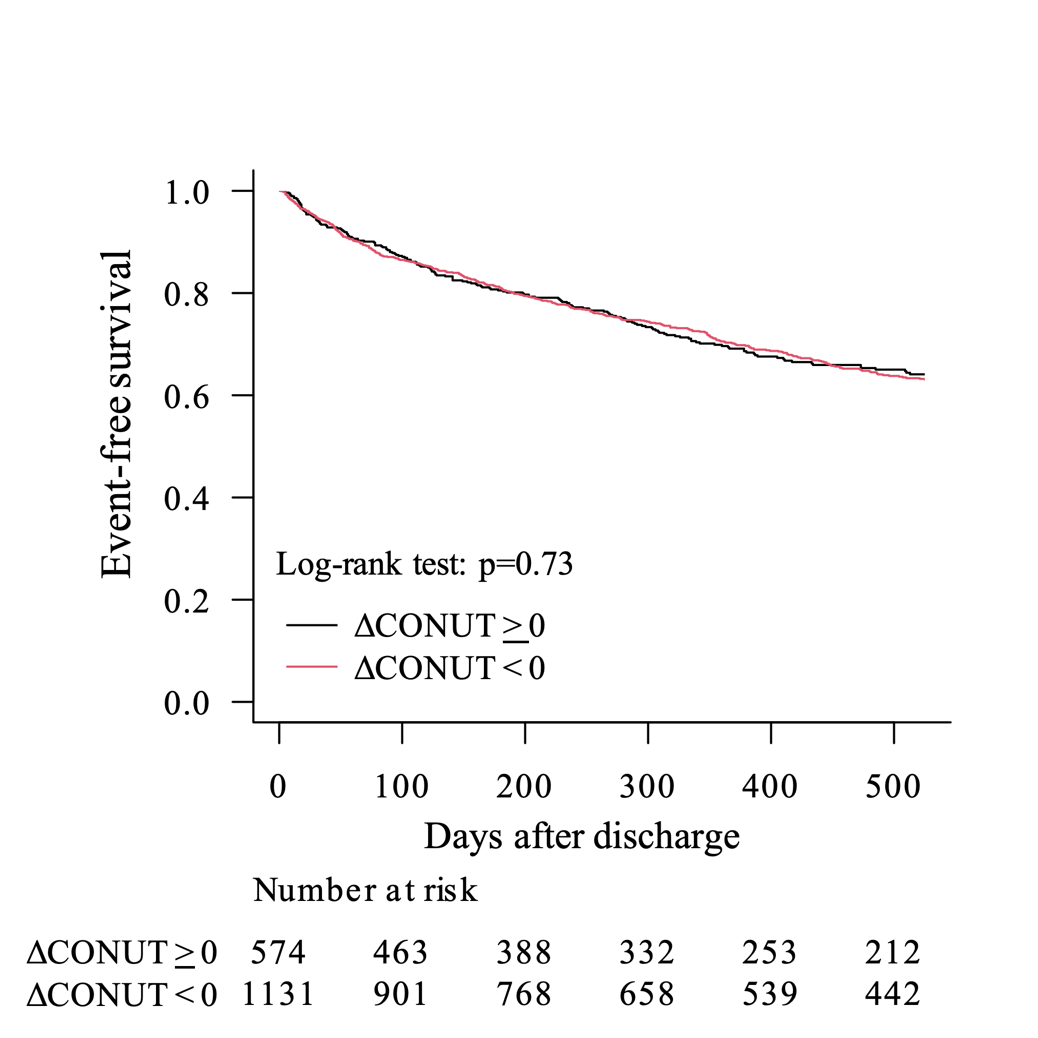
**

**Figure S5. Composite outcome after discharge among the 4 subgroups categorized according to the CONUT scores at admission and discharge in patients with HFrEF or HFpEF**
